# Self-Assembled Wiggling Nano-Structures and the Principle of Maximum Entropy Production

**DOI:** 10.1038/srep08323

**Published:** 2015-02-09

**Authors:** A. Belkin, A. Hubler, A. Bezryadin

**Affiliations:** 1 Department of Physics, University of Illinois at Urbana-Champaign, 1110 W. Green Street, Urbana, IL

## Abstract

While behavior of equilibrium systems is well understood, evolution of nonequilibrium ones is much less clear. Yet, many researches have suggested that the principle of the maximum entropy production is of key importance in complex systems away from equilibrium. Here, we present a quantitative study of large ensembles of carbon nanotubes suspended in a non-conducting non-polar fluid subject to a strong electric field. Being driven out of equilibrium, the suspension spontaneously organizes into an electrically conducting state under a wide range of parameters. Such self-assembly allows the Joule heating and, therefore, the entropy production in the fluid, to be maximized. Curiously, we find that emerging self-assembled structures can start to wiggle. The wiggling takes place only until the entropy production in the suspension reaches its maximum, at which time the wiggling stops and the structure becomes quasi-stable. Thus, we provide strong evidence that maximum entropy production principle plays an essential role in the evolution of self-organizing systems far from equilibrium.

Classical thermodynamics offers elaborated theoretical instruments to study behavior of systems that are in or near equilibrium. But often objects and processes which we deal with in everyday life, such as living biological organisms or weather events, are far from equilibrium. Therefore, the search for principia governing nonequilibrium systems is of great interest.

The notion of entropy in equilibrium states and its production in nonequilibrium processes not only form the basis of modern thermodynamics and statistical physics, but have also been at the core of various philosophical discussions concerned with the evolution of the world, the course of time, etc. There is a general agreement on how the entropy of a nonequilibrium system should evolve, postulated by the second law of thermodynamics. At the same time, the question how entropy production changes with time is still extensively discussed. So-called the maximum entropy production principle (MEPP)^1^,^2^,^3^ was proposed in the middle of 20^th^ century^4^,^5^,^6^,^7^ and may be viewed as the natural generalization of the Clausius-Boltzmann-Gibbs formulation of the second law. According to MEPP, a nonequilibrium system evolves such as to maximize its entropy production under present constraints. Proponents of this principle demonstrated that it can be used as a powerful tool to solve various environmental^8^,^9^,^10^, biological^11^,^12^ and physical problems^13^,^14^,^15^,^16^. It is interesting to notice that there are publications attempting to disprove MEPP^17^,^18^,^19^,^20^,^21^,^22^. However, the most recent review^23^ argues that all these claims seemingly disproving MEPP are based on experiments, which do not belong to the sphere of applicability of the principle.

Frequently, thermodynamic systems driven out of equilibrium have a tendency for symmetry breaking. This includes pattern formation^24^, spontaneous self-assembly of particles either dispersed in colloids^25^,^26^,^27^, suspended at the air-fluid^28^,^29^ or fluid-fluid interfaces^30^. One of the best-known examples of symmetry breaking is the emergence of Rayleigh–Bénard (RB) convection cells^31^ in a layer of oil placed on a hot plate. Ordering that is present in the RB cells *reduces* the oil entropy. At the same time, convective flows associated with the RB structures *increase* the heat transfer rate, leading to a greater entropy production of the entire system, which includes the hot plate and the environment. It is unclear under which conditions nonequilibrium systems develop a new order. Yet, it is plausible to hypothesize that MEPP might be one of the main guiding principles for not only the evolution of biological species, but their appearance on Earth due to the strongly nonequilibrium energy distribution created by the Sun^32^,^33^. Nonequilibrium conditions occur because the Sun supplies photons with energies of many thousands of Kelvins, which is much larger than the temperature of the surface of the Earth. Taking into account these facts, we believe that investigations of the MEPP applicability for self-organizing systems is a very promising direction of research, from the viewpoint of both the substantiation of the MEPP and for better understanding and predicting behavioral and evolutionary trends in nonequilibrium systems.

In this work, we investigate possible evolution paths of an electrorheological (ER) fluid^34^ exposed to a strong electric field (E-field) towards an attractor state. Our setup provides means to study nonequilibrium processes *quantitatively*. In particular, it enables precise measurements of generated Joule heating (the precision is limited by a very small and uncertain fraction of internal work done by the voltage source that is used to create order in the system of nanotubes). As a result, entropy production in the fluid, which is directly proportional to the Joule heating, can be correlated with the process of self-assembly of carbon nanotube (CNT) chains occurring under the effect of the applied E-field^35^,^36^,^37^,^38^,^39^. We demonstrate that in such complex systems as ER fluids the state with maximum entropy production acts as an attractor.

## Results

When the ER fluid is driven far from equilibrium by a strong E-field, it may exhibit a spontaneous collective behavior, such as emergence of avalanches and chain formation. Previous studies were suggestive that these processes lead to maximization of the entropy production rate^40^. Here we use a different suspension of conducting particles and observe that the ER fluid evolves towards quasi-stable states characterized by the maximum (or near-maximum) rate of entropy increase in a wide range of excitation parameters. The evolution usually proceeds in two phases: an “avalanche” phase followed by a “stable” phase, which onsets when the entropy generation reaches its pre-determined maximum. We discovered that close to the end of the avalanche phase self-assembled dissipative structures can start to *wiggle, i.e.* exhibit quasi-periodic motion and deformation (for the video containing details see the supplementary online material). Due to a visual resemblance to a living organism, we term such states as a “bug”. Interestingly, the bug, which takes energy from the biased electrodes, halts its motion as soon as the entropy production in the ER fluid *reaches* the maximum.

### Experiment details

**Figure 1:**
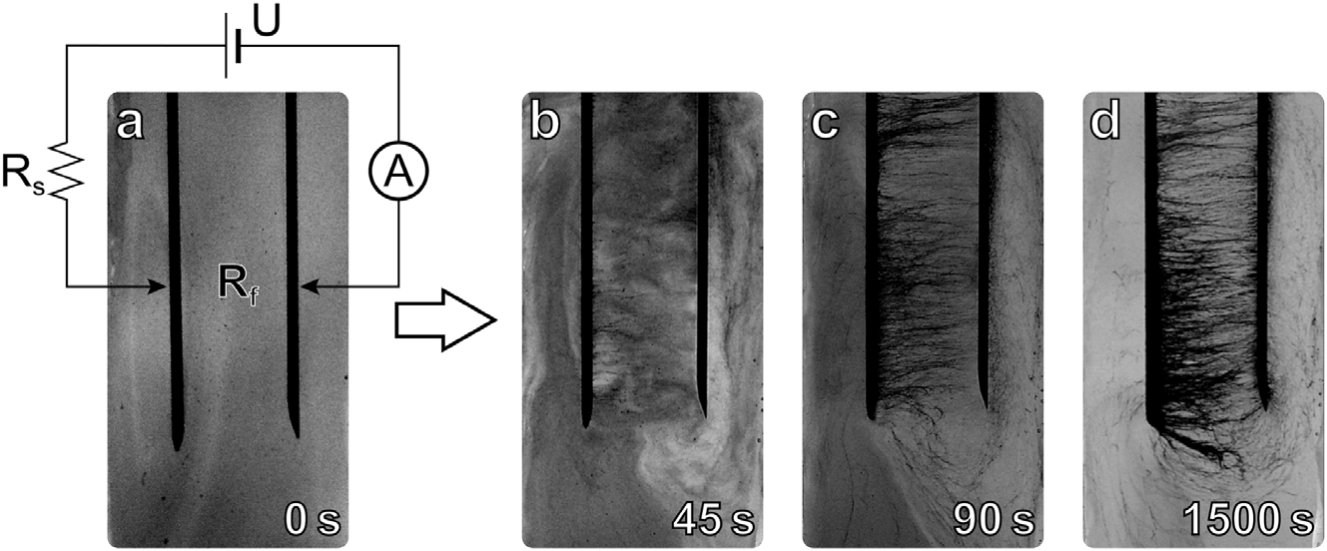
Consecutive snapshots of the sample illustrating the formation of nanotube chains. The distance between electrodes is 1 cm, applied voltage is *400* *V*, and the series resistor is *100* *MOhm*. Panel (a) demonstrates the photograph of the ER fluid before the voltage is applied and the schematic of the experimental setup. The following photographs are taken after (b) *t* = *45* *s*, (c) *t* = *90* *s* and (d) *t* = *1500* *s* of interaction with E-field.

The fluid is comprised of electrically conducting either of single- (SES Research, outer diameter <*2* *nm*, length *5*–*15* *μm*) or multi-wall carbon nanotubes (Alfa Aesar, outer diameter *3*–*24* *nm*, length *0.5*–*5* *μm*) suspended in an insulating non-polar solvent (toluene). The field is generated between a pair of metallic electrodes immersed in the ER fluid and connected to a voltage source in series with a commercial resistor. The measurement setup involves a voltage source connected in series with a resistor, ammeter, and two cylindrical stainless steel electrodes submerged into the sample (Fig. 1a). The diameter of the electrodes is *0.7* *mm*, the spacing between them is *10* *mm*, and the depth of immersion into the fluid is over *10* *mm*. The nanotube concentration is varied between *0.05* and *0.2* *mg/ml*. This is significantly below the percolation threshold, *i.e.* the current in the circuit is negligibly small when the nanotubes are not aligned with each other. However, all the concentrations we use allow a nontrivial self-assembly: when the tubes form chains, the electric current, *I = I*(*t*), can reach and even exceed *U/2R_s_*, where *R_s_* is the series resistance and *U* is the applied voltage. Since the current depends on the presence of continuous nanotube chains and electro-convection, we can use it as a measure of the order in the suspension.

Prior to each experiment, the nanotubes in the fluid are dispersed by means of sonication, ensuring that the initial resistance of the suspension, *R_f_*(*t* = *0*), exceeds *R_s_* by at least *3* orders of magnitude. The time *t* is measured from the moment when the voltage is turned on. During the measurement, the value of the series resistor is fixed, while the resistance of the fluid is changing owing to the formation of CNT chains. We use a precision electrometer (Keithley 6517B) to set the voltage and to measure the current in the circuit. The power dissipated in the fluid, *P_f_*, can be deduced from the equation *P_f_*(*t*) = *I*(*t*)[*U-I*(*t*)*R_s_*], where *I*(*t*)*R_s_* is the voltage drop on the resistor.

### Chain growth

Snapshots of the system evolution under the influence of a strong electric field are demonstrated in Figure 1. Before the voltage is turned on, the carbon nanotubes are homogeneously dispersed in the fluid, providing its uniform grey coloring (Fig. 1a). Soon after the bias is applied, we observe a turbulent motion of the fluid. This motion is caused by the electro-convection, or the “shuttling” effect^40^, see Fig. 1b, when the electric charge is transported from one electrode to the other by small but visible clusters of charged particles. In parallel with the charge transfer process, chains of nanotubes begin to grow. Characteristically, the growth starts from the positive electrode (left electrode in Fig. 1) and proceeds towards the negative one, which is grounded in our setup. The process of self-organization is not always monotonic, *i.e.* the chains can get destroyed, what represents an *avalanche*. This evolution stage is called the “avalanche” phase due to the fact that they frequently, but not necessary, occur here. The reasons for the avalanches have been clarified in the model presented in Ref. 40.

At a certain value of the power dissipated in the fluid, the system transitions to a stable state, which is characterized by termination of a turbulent motion and formation of a steady connection between the electrodes (Fig. 1c). We term this stage of the system evolution a “stable” phase. The stable phase begins at time *t_0_* and continues as long as the experiment goes on. During that phase, the chain breaking events can no longer be detected visually. Quite the contrary, more and more nanotubes are attracted to the electrodes, making the chain network larger and denser, while the rest of the fluid becomes clearer (Fig. 1d). As will be discussed later, the stabilization coincides in time with the moment *t_0_* when the power dissipated in the fluid reaches its highest possible value, determined by the applied voltage and the value of the series resistor.

Now we want to comment on the linearity of the process. Before the maximum entropy production is achieved, the process is nonlinear. Chains of nanotubes are formed under the effect of strong electric field and get destroyed by fluctuations, gravity, Coulomb forces, and, possibly, by some local Joule heating inside electrically conducting chains. When the maximum of the entropy production is achieved, we notice that the cloud formed by nanotubes and attached to the electrodes is rather stable. Therefore, small variations of the bias voltage do not change the resistance of the structure in this regime. Hence, the charge flow is approximately a linear function of the applied electric field. On the other hand, if the voltage is reduced significantly, the gravity and fluctuations cause a certain amount of degradation of the self-assembled cloud of nanotubes. In case of strong variations of the applied voltage the response is nonlinear. Generally speaking, when we emphasize that the applied electric field is strong we mean that it is sufficient to change the organization of the nanotubes and thus it can change the resistance of the fluid. The self-assembly in nonequilibrium systems is inherently nonlinear process.

## Discussion

The chain formation mechanism can be explained by means of simple dipole-dipole interaction. Indeed, the applied E-field polarizes nanotubes, making them act as electric dipoles. Since a dipole moment aligns along the field, all nanotubes tend to be in parallel with each other. Such orientation of dipoles creates an attractive force between them, when the positively charged end of one nanotube connects to the negatively charged end of a neighboring nanotube. Thus, chains of nanotubes start to grow.

Our setup has an important property that we want to emphasize: Joule heating within the fluid, generated by the current flowing through CNT chains, has a maximum^40^: *P_max_* = *U*^*2*^*/4R_s_*. This maximum is achieved *only* when the nanotubes organize in such a way that their collective resistance matches the series resistance, *i.e.* when *R_f_* = *R_s_*. The above condition could be thought of as a manifestation of the maximum power transfer theorem. However, it should be stressed that this theorem does not predict any particular direction of the evolution for self-organized systems included into the circuit, as is the case in our experiments. Thus, it cannot be used to predict that the power generated in the self-assembled fluid should achieve its maximum in the process of evolution initiated by the applied strong electric field.

The temperature increase of the ER fluid, Δ*T*, depends on the applied voltage, duration of the experiment and on the suspension's resistance, mass and specific heat. Simple estimates show that even if the system was thermally isolated, the maximum Δ*T* for the characteristic parameters of our experiment should not exceed *0.15* *K*. It constitutes only *0.05%* of the fluid temperature. In the real situation there is a heat flow from the fluid to an environment, and therefore Δ*T* is even smaller. To confirm this estimate, we have measured the temperature of the fluid during the self-assembly process and detected no temperature increase to an accuracy of *0.2* *K*. Therefore, we take *T_f_* ≈ *T* = *constant*, where *T and T_f_* is the ambient and the fluid temperature, correspondingly.

The entropy production in the fluid is calculated as the power dissipated in the fluid divided by the environment temperature, *i.e.* *dS_f_/dt* = *P_f_/T*. Thus, MEPP in this system corresponds to the condition of the maximum power dissipated in the fluid, which is achieved when *R_f_* = *R_s_*.

**Figure 2:**
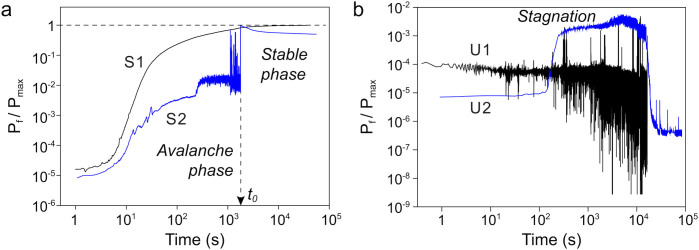
Normalized power dissipation in the fluid as a function of time, *t*. Concentration of nanotubes is *0.075* *g/l*. (a) Complete evolution: curve S1 (black), *R_s_* = *10* *MΩ*, *U* = *75* *V*; curve S2, *R_s_* = *10* *MΩ*, *U* = *325* *V* (blue). The time *t_0_* is the time when the maximum possible dissipated power is achieved. (b) Incomplete evolution: curve U1, *R_s_* = *100* *MΩ*, *U* = *5* *V*; curve U2, *R_s_* = *2* *MΩ*, *U* = *300* *V*.

Let us now turn to Figure 2, which demonstrates how the power dissipated in the fluid changes under various experimental conditions. In general, we distinguish two evolution scenarios. When the system evolves according to the first scenario, which we call the “*complete evolution*”, *P_f_* reaches *P_max_*. At the initial stage of such evolution and before *P_max_* achieved, the average power increase may either proceed smoothly (curve S1 in Fig. 2a) or be accompanied by spontaneous avalanches (curve S2 in Fig. 2a). The general trend is that overall the dissipation tends to increase.

From multiple visual observations we identify that the moment at which *P_f_* matches *P_max_* coincides with the beginning of the stable phase. During the system evolution in the stable phase, dissipation changes slowly over time and *P_f_* stays on the order of *P_max_* for the remaining and the longest part of the experiment (note the logarithmic scale in Fig. 2). Similarly to Ref. 40, no avalanches are observed in the stable phase of a complete evolution. Departure of *P_f_* from *P_max_* at *t* > *t_0_* corresponds to a gradual decrease of *R_f_* below *R_s_*, which is caused by the thickening of the CNT chains, and is not a result of their destruction.

When the system evolves according to the second scenario, termed “*incomplete evolution*”, *P_f_* does not reach *P_max_* (Fig. 2b), and CNT precipitate. In the early phase, the chains may start to form, which together with the electro-convection leads to an increasing *P_f_*. Later on, this growth slows down and the system eventually enters a stagnation phase. At the next, the final phase, the system “dies”: nanotubes precipitate and the dissipated power drops to a value many orders of magnitude below *P_max_*. There are three feasible reasons for the incomplete evolution. First, when an applied E-field is too weak (curve U1 in Fig. 2b), the “destructive” forces, such as gravity and thermal vibration of nanotubes, are able to overcome the attractive dipole-dipole forces, which results in uncorrelated diffusion of the nanotubes and, later, to their slow, but irreversible precipitation. Incomplete evolution can also be caused by a too strong electric field (curve U2 in Fig. 2b), which creates intense turbulent flows in the fluid, precluding formation of stable chains. Moreover, when the first chains, connecting two electrodes, are formed, strong E-fields induce high local electric currents heating up these chains and eventually burning them out. And finally, at low concentrations of CNT, the system is not able to build a stable network of chains that matches the series resistance.

**Figure 3:**
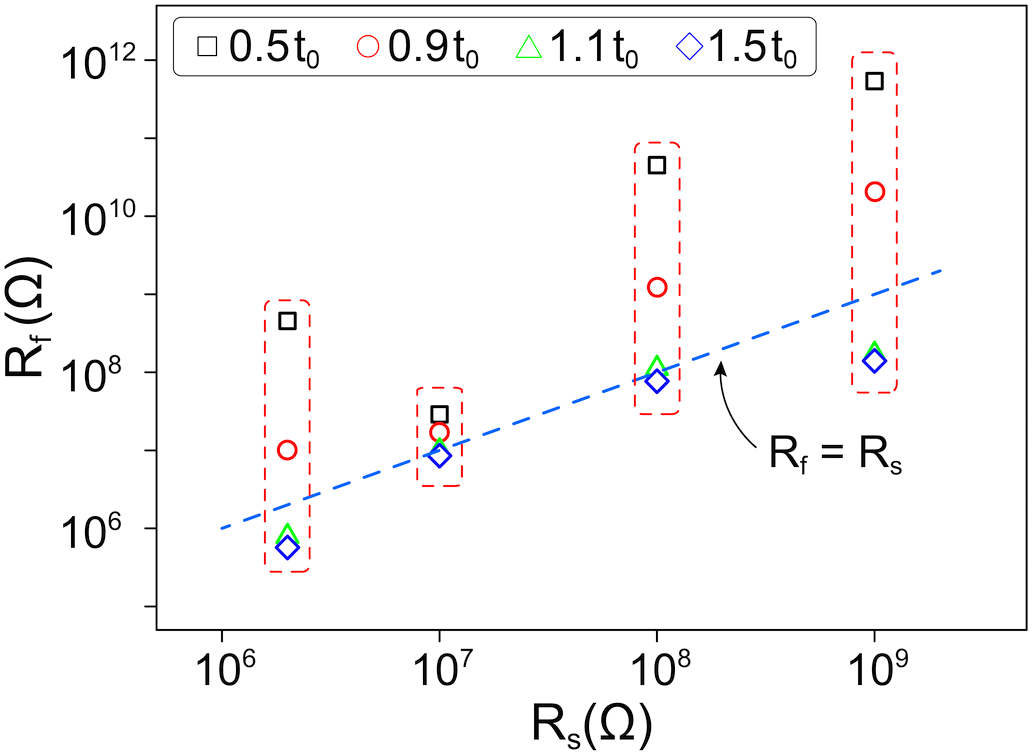
The resistance of nanotube chains formed in the fluid at different series resistances. For each *R_s_* four values of *R_f_* are shown, measured at different times *t*. Blue diamonds represent the longest evolution time. Applied voltage is *150* *V*, concentration of nanotubes is *0.07*5 *g/l*. The blue dashed line corresponds to *R_f_* = *R_s_*, *t_0_* is the time when *P*(*t_0_*)/*P_max_* = *1*.

Despite the number of factors impeding the formation of CNT chains, the complete evolution takes place in a surprisingly wide range of experimental parameters. To study the ability of the system to adjust its resistance to reach *P_max_*, we performed a number of experiments in which we fixed the applied voltage *U* and varied the series resistance *R_s_*. The results of these measurements are plotted in Figure 3. As one can see, *R_f_* tends towards *R_s_* with time. This finding is especially remarkable since the series resistance is varied across three orders of magnitude, whereas all other parameters, namely the concentration of nanotubes and the applied voltage, are kept the same.

**Figure 4:**
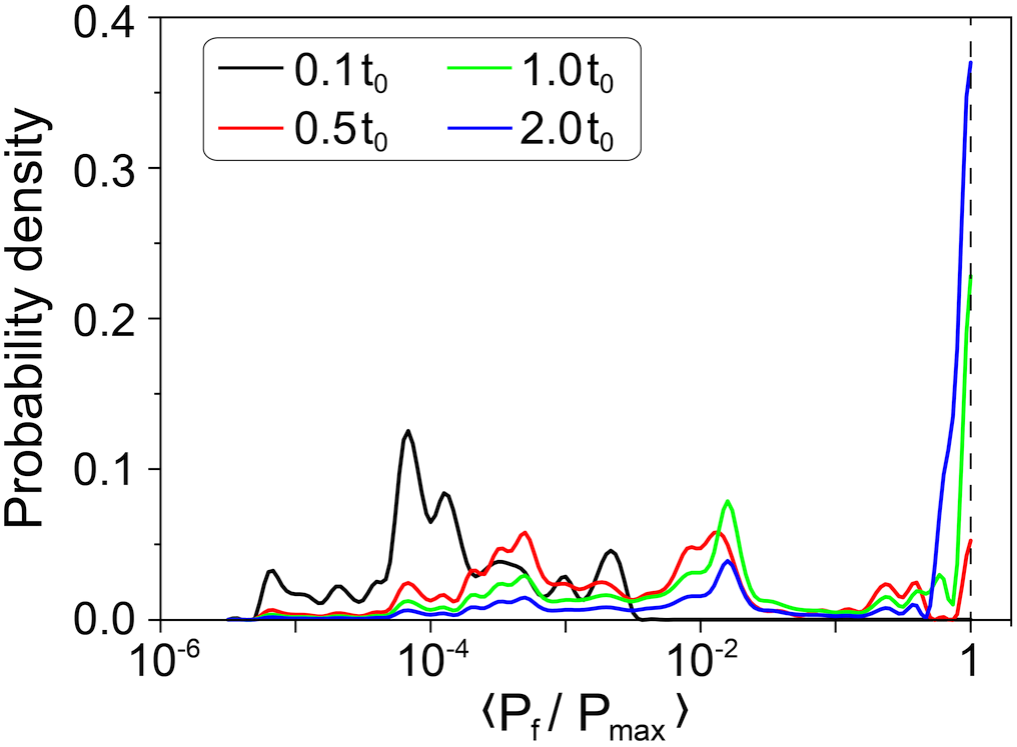
Probability density function of the normalized power dissipated in the fluid. These curves are calculated using the measurements of the type shown in Fig. 2. The probability distributions are calculated for the following time intervals: 0 < *t* < *0.1t_0_*, 0 < *t* < *0.5t_0_*, 0 < *t* < *1.0t_0_* and 0 < *t* < *2.0t_0_*. The total ensemble consists of *438820* measured points, corresponding to *ten* independent evolution curves, in which *R_s_* and *U* had different values.

The tendency of the system to maintain dissipation that is close to its maximum is demonstrated in Figure 4. Here, the probability density function of the dissipated power averaged over *10* measurements is plotted for several moments of time during a complete evolution. In the beginning of the avalanche phase, *i.e.* at *t* = *0.1t_0_*, relatively low Joule dissipation values have the highest probability. Later on, the distribution shifts towards the states with higher dissipation level, and at *t* = *0.5t_0_* we already observe three distinct peaks. During the stable phase of the evolution, the probability peak that corresponds to the maximum dissipation continues to grow, while the amplitudes of all other peaks diminish. This result supports our conclusion that throughout the evolution the system tends to self-organize in such a way that the dissipation of the injected energy proceeds most efficiently. Therefore, the entropy production in the fluid proceeds at the maximum rate for a given set of constraints (applied voltage and the series resistor). Once the maximum is achieved, the evolution processes slow down, giving rise to the peak in the vicinity of <*P_f_/P_max_*> = *1*. Note that if we consider the entire system that includes the fluid and the resistor, then the maximum of the entropy production is never achieved, since it would correspond to *R_f_* = *0*.

**Figure 5:**
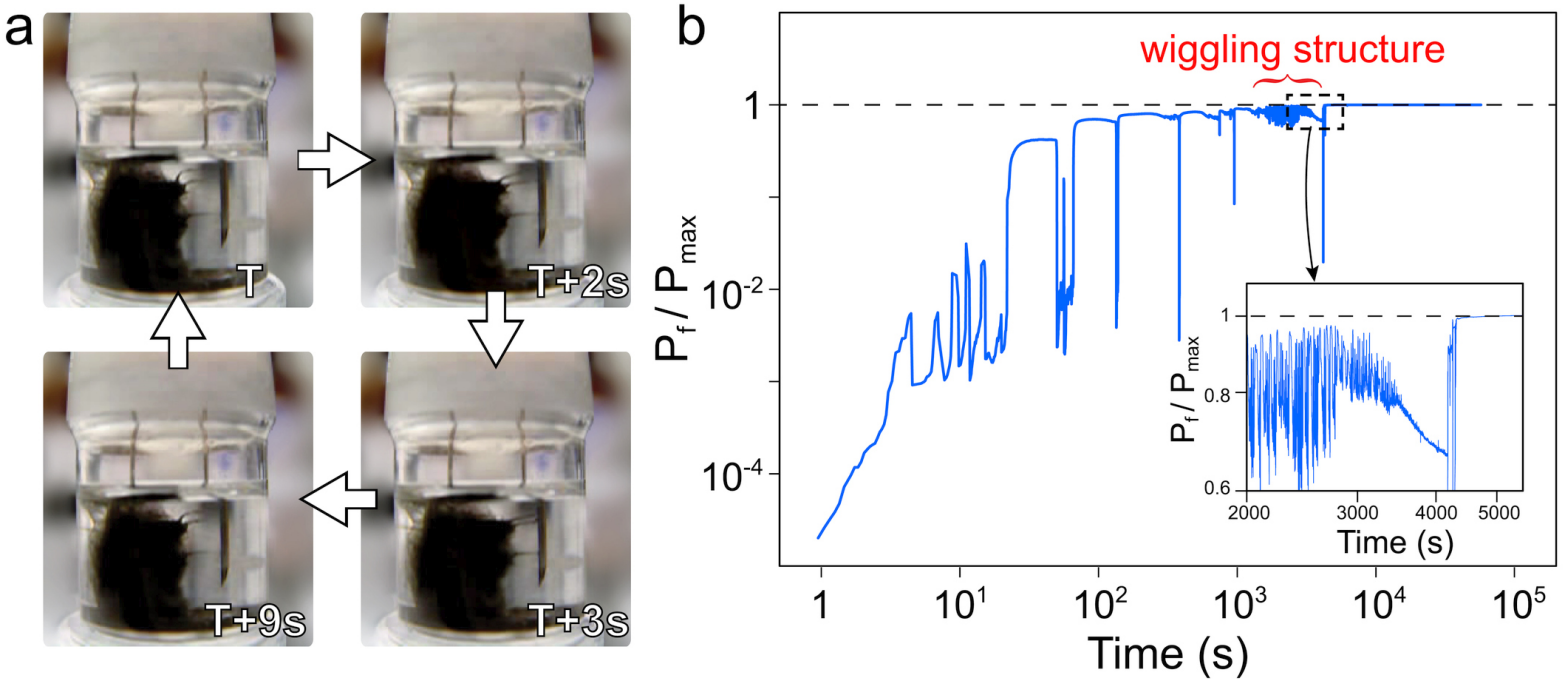
Motion of the CNT “bug” under the influence of dc electric field. Panel (a) illustrates positions of the “arms” of the bug when they are retracted (*t* = *T*), approaching (*t* = *T+2* *s*), touching (*t* = *T+3* *s*) and again retracted (*t* = *T+9* *s*) from the right electrode. Here *T~2500 s*. The movie showing the motion of the selfassembled wiggling structure is contained in Supplementary Information online. Panel (b) demonstrates the time dependence of the normalized power dissipated by the bug. Inset: a segment of the time dependence of the normalized power, corresponding to the phase when the bug is fully developed and exhibits the arm motion. At *t~4500 s* the stable phase begins.

Occasionally, towards the end of the avalanche phase, nanotubes form a dissipative pattern that exhibits quasi-periodic collective motion and deformation. Figure 5 shows an example of such a dissipative structure (CNT bug) that self-assembled around one of the electrodes (see video in Supplementary Information). A few chains of nanotubes form thick CNT “arms” that protrude out of this bug and extend towards the opposite electrode (Fig. 5a, 2s). Their motion forces the cloud to displace, causing its slight deformation. When the arms touch the electrode, they remain in contact with it for a short period of time (Fig. 5a, 3s), and then retract (Fig. 5a, 9s). Subsequently, the process repeats again (Fig. 5a). This wiggling of the dissipative structure is surprising since the applied voltage is constant in time. The extension and retraction of several CNT arms is almost synchronous. The motion of the CNT bug is repeated and can be characterized as quasi-periodic, up until the stabilization transition when the system enters the stable evolution phase.

In the course of the CNT bug formation, the initial rapid growth of the dissipated power is accompanied by relatively large avalanches (Fig. 5b). Once the body and the arms of the CNT bug have formed, the dependence *P_f_/P_max_*(*t*) starts to jitter near the maximum, but does not actually reach it (inset in Fig. 5b). This jittering correlates with the arms' motion (see Fig. 5a), namely the power increases when they touch the other electrode (right electrode in Fig. 5a), and decreases as soon as they retract. After approximately an hour of this wiggling behavior, a transformation to a state with the maximum Joule heating in the nanotube structure takes place. At that moment the center of mass of the cloud of nanotubes shifts to the central position between the electrodes. The connection of the cloud to the left electrode remains stable and a similar stable connection with the right electrode gets established. The bug halts its motion, and *P_f_* remains near *P_max_* for as long as the experiment is continued. As time goes by, the center of mass of the cloud slowly shifts closer to the bottom of the container, apparently due to gravitational forces. Yet, the connection of the cloud to both electrodes remains stable, and the dissipated power continues to be at its maximum.

To sum up, we have studied the behavior of the electrorheological fluid under the influence of a strong electric field. Two types of the evolution have been observed: complete, max(*P_f_*(*t*)) = *P_max_*, and incomplete, max(*P_f_*(*t*)) < *P_max_*. In the course of complete evolution, the total resistance of self-assembled carbon nanotube structure tends to a value which maximizes energy dissipation in the structure. This fact reflects the realization of the maximum entropy production principle. Moreover, the self-assembled dissipative structures can start to wiggle, thus resembling a self-created living organism. We find that the system is dynamic if its resistance is greater than the series resistor, and it becomes static when the impedance matching condition, *R_f_* = *R_s_*, is achieved. Our conclusions apply to systems subjected to external forces and gradients, which are sufficiently strong so that the behavior is nonlinear and the self-assembly cannot be prevented by external or internal perturbations. On the other hand, the applied forces and/or gradients should not be too strong. Otherwise the generated heating might destroy self-assembled patterns.

## Author Contributions

A.H. wrote the grant that made this work possible. A. Bez. and A. Bel. designed the experiment. A. Bel. performed the measurements. All authors contributed to the analysis and the writing of the manuscript.

## Supplementary Material

Supplementary Information Motion of the CNT
